# Validation of the International League Against Epilepsy (ILAE) Risk of Bias Tool against the Newcastle–Ottawa Scale in epilepsy research

**DOI:** 10.1002/epi.70183

**Published:** 2026-03-08

**Authors:** Churl‐Su Kwon, Ali Rafati, Nathalie Jette, Charles R. Newton

**Affiliations:** ^1^ Department of Neurology, Epidemiology, Neurosurgery and the Gertrude H. Sergievsky Center Columbia University Irving Medical Center New York New York USA; ^2^ Department of Psychiatry University of Oxford Oxford UK; ^3^ Department of Neurology Johns Hopkins University Baltimore Maryland USA; ^4^ Department of Neurology University of Calgary Calgary Alberta Canada

**Keywords:** bias, epilepsy, International League Against Epilepsy, reproducibility of results, risk assessment

## Abstract

**Objective:**

Systematic reviews and meta‐analyses (SRMAs) are critical for synthesizing evidence and guiding clinical and public health decision‐making. This study aims to evaluate the reliability, validity and reproducibility of the International League Against Epilepsy (ILAE) Commission on Epidemiology Risk of Bias Tool by comparing it against the Newcastle–Ottawa Scale (NOS) to inform whether the ILAE tool may serve as a valid alternative in epilepsy‐focused evidence syntheses.

**Methods:**

This study was planned a priori on three consecutive SRMAs. We assessed 54 observational studies included in these SRMAs focused on psychiatric comorbidities in persons with epilepsy. Eligible studies had ≥30 participants per group and validated criteria for diagnosing epilepsy and psychiatric conditions. Two independent raters scored all studies using both tools. The ILAE tool comprises six specific domains: (1) Source of Study Population; (2) Completeness (Sensitivity) of Epilepsy Case‐Finding; (3) Sensitivity of Comorbidity Determination; (4) Accuracy of Epilepsy Diagnosis; (5) Accuracy of Comorbidity Diagnosis; and (6) Representativeness of Study Sample. Test–retest reproducibility used intraclass correlation coefficient (ICC). Correlation used Spearman's rho. Agreement used weighted kappa. Bland–Altman analysis reported mean difference.

**Results:**

There was a strong positive correlation between NOS scores and ILAE ratings (Spearman's rho = 0.80, 95% confidence interval [CI] 0.68–89, *p* < 0.001). Cohen's weighted kappa was 0.68 (95% CI 0.37–0.92, *p* < 0.001). Bland–Altman mean difference was 0.09 with limits from −0.48 to 0.67, showing good agreement between tools. The ILAE tool showed excellent test–retest reproducibility (ICC 0.90, 95% CI 0.83–0.94).

**Significance:**

The ILAE tool demonstrated strong reliability and substantial agreement with the NOS while offering epilepsy specific rigor in diagnostic accuracy, sensitivity, case finding and representativeness. The ILAE tool offers a reliable, conceptually relevant, field‐specific alternative for quality assessment in epilepsy SRMAs.

**Key points**

1. Strong correlation between the International League Against Epilepsy (ILAE) tool and the Newcastle–Ottawa Scale (NOS) (Spearman's rho = 0.80).
2. Substantial agreement in identifying low‐quality studies (Cohen's weighted kappa = 0.68).
3. Excellent 6‐month test–retest reproducibility of the ILAE tool (intraclass correlation coefficient [ICC] = 0.90).
4. The ILAE tool adds epilepsy‐specific rigor in diagnostic accuracy and completeness of epilepsy case ascertainment.
5. The ILAE risk of bias tool is a reliable, field‐specific alternative for epilepsy systematic reviews and meta‐analyses.

## 1 INTRODUCTION

Systematic reviews and meta‐analyses (SRMAs) are critical for synthesizing evidence in epilepsy research and guiding clinical and public health decision‐making. The validity of SRMA findings depends in large part on the quality of the primary studies they include and the rigor with which that quality is assessed. Although the Newcastle–Ottawa Scale (NOS) is one of the most widely used tools for evaluating the risk of bias in observational studies,^1^ it was designed for general epidemiological use and does not address key methodological issues specific to epilepsy research. These include epilepsy case definition and diagnostic accuracy, completeness of epilepsy case ascertainment, and rigor of psychiatric comorbidity determination, which are often only indirectly captured by general‐purpose tools such as the NOS.

To address this limitation, the International League Against Epilepsy (ILAE) Commission on Epidemiology developed a risk of bias tool tailored specifically to epilepsy research.^2^ This tool includes domains such as the accuracy of epilepsy and comorbidity diagnoses, completeness of case ascertainment, and the representativeness of study samples, features that are often assessed only indirectly by general‐purpose tools like the NOS. The ILAE tool provides a structured, epilepsy‐specific framework that facilitates consistent appraisal of diagnostic rigor, case‐finding, and representativeness across heterogeneous study designs commonly encountered in epilepsy SRMAs.

Although the ILAE tool was designed to improve the quality assessment of epilepsy studies, its performance has not yet been evaluated against existing standards. This study aims to assess the reliability and validity of the ILAE Risk of Bias Tool by comparing it to the NOS across a sample of observational studies included in recent SRMAs focused on psychiatric comorbidities in people with epilepsy.^3^, ^4^, ^5^ The findings will inform whether the ILAE tool may serve as a valid alternative to the NOS in future epilepsy‐focused evidence syntheses.

## 2 METHODS

### 2.1 Study design and objective

This methodological validation study was planned a priori as a predefined ancillary analysis conducted alongside three consecutive SRMAs; however, it was not formally preregistered. This methodological study aimed to evaluate the reliability and validity of the ILAE Commission on Epidemiology Risk of Bias Tool by comparing it against the established NOS. The ultimate goal is to provide a validated, epilepsy‐specific standard endorsed by the ILAE for assessing risk of bias in future systematic reviews and meta‐analyses.

### 2.2 Data sources and study selection

We assessed studies included in the three SRMAs: one on psychiatric comorbidities in persons with epilepsy (PwE) compared to controls,^3^ another on multi‐psychiatric comorbidity in PwE,^4^ and a third on suicide‐related outcomes in PwE.^5^ Eligible studies were original, peer‐reviewed observational studies (cohort or cross‐sectional), with at least 30 participants per group, and utilized validated criteria for diagnosing epilepsy and psychiatric conditions. After removal of duplicates, 54 unique studies were included.

### 2.3 Quality assessment tools

The NOS was selected as the comparator instrument because it represents the most commonly applied risk‐of‐bias tool in observational epilepsy SRMAs, thereby reflecting prevailing methodological practice in the field. In the absence of a consensus gold standard for epilepsy‐specific risk‐of‐bias assessment, comparison against NOS allows benchmarking of the ILAE tool relative to current standards used in published evidence syntheses. The NOS evaluates three domains: selection, comparability, and outcome/exposure assessment. It produces a score from 0 to 10 for cross‐sectional and from 0 to 9 for cohort studies. We categorized NOS scores as High (7–9/10), Moderate (4–6), and Low (0–3), consistent with thresholds used in prior systematic reviews and recommendations from recent methodological literature.^6^, ^7^ The ILAE tool comprises six domains specifically tailored to epilepsy epidemiology (Table 1): (1) Source of Study Population; (2) Completeness (Sensitivity) of Epilepsy Case‐Finding; (3) Sensitivity of Comorbidity Determination; (4) Accuracy of Epilepsy Diagnosis; (5) Accuracy of Comorbidity Diagnosis; and (6) Representativeness of Study Sample. Final ILAE judgments were collapsed into four levels: High, Good, Fair, and Poor, with the lowest‐scoring domain determining the overall rating, as per recommendations from the ILAE^2^ (Table 1).

**TABLE 1:** Structure of ILAE Risk of Bias Tool.

**Report type**
1 = Original research on population of ≥30 people with epilepsy or unprovoked seizure and ≥30 people without epilepsy
2 = Meta‐analysis
3 = Other (would exclude)
**Psychiatric comorbidity measured?**
*Relevant comorbidities of epilepsy are limited to psychiatric conditions (includes neurodevelopmental conditions e.g., autism, intellectual disability). Measures of risk include rates, proportions, and relative comparisons (e.g., RR, OR) or can be calculated*
0 = No
1 = Yes
**Proceed with rating quality?**
0 = No (does not meet criteria above)
1 = Yes (If report type #1 or 2 and psychiatric multimorbidity measured)
**Study‐level risk‐of‐bias rating (the lowest ranking score from any category will be the “calculated rating” for the study)**
*Study type*
1 = Incidence (prospective or retrospective cohort study)
2 = Prevalence (cohort or cross‐sectional)
3 = Case–control
4 = Other or mixed
*Source of study subjects*
1 = Population‐based
2 = Clinic‐based, broad representation (e.g., all people with epilepsy in the practice)
3 = Clinic‐based, limited representation (e.g., only women with epilepsy, people with JME, people who had epilepsy surgery)
4 = Other, *poorly defined, or highly skewed subject*
**Completeness (sensitivity) of epilepsy case‐finding in study population**
*Reviewer's judgment of the sensitivity of methods of epilepsy diagnosis: completeness of epilepsy case ascertainment in population‐based studies of epilepsy incidence or prevalence*:
0 = Not population‐based/Not applicable. Clinic‐based study where subjects are selected based on epilepsy diagnosis
1 = High – nearly all cases. Methods appear likely to find nearly all cases of epilepsy in population (or sampled population)
2 = Good – most. Screening methods appear likely to ascertain most (approx. 70%–84%) cases in population
3 = Fair – majority. Screening methods appear likely to ascertain majority (approx. 50%–69%) of cases in population
4 = Poor – minority. Screening methods appear unlikely to ascertain majority of cases in population, OR information published is insufficient to assess
**Sensitivity of comorbidity determination**
*Reviewer's judgment of the sensitivity of methods of comorbidity ascertainment*
1 = High – nearly all cases evaluated and classified. Comorbidities appear likely to be recorded in nearly all (≥85%) cases in the study population
2 = Good – most cases evaluated and classified. Comorbidities appear likely to be recorded in most (70%–84%) cases in the study population
3 = Fair – majority of cases evaluated and classified. Comorbidities appear likely to be recorded in the majority (50%–69%) of cases in the study population
4 = Poor – majority not classified or insufficient. Comorbidities appear unlikely to be recorded in the majority of cases in study population, OR unable to determine
**Accuracy of epilepsy diagnoses**
*Reviewer's judgment of the accuracy of diagnoses of epilepsy*:
1 = High – specialist diagnosis or valid structured interview; ILAE definition Cases are diagnosed by specialist clinician (i.e., w/ neurologic training), AND ILAE case definition applied
2 = Good – mainly non‐specialist clinicians. Cases are diagnosed by non‐specialist clinician, OR minor deviation from ILAE case definition, OR all or substantial proportion of cases diagnosed based on ICD codes
3 = Fair – self‐report; non‐clinical sources. All or substantial proportion of cases diagnosed based on self‐report or non‐clinical sources with specified criteria judged to have fair (>50%) positive predictive value (PPV)
4 = Poor – ill‐defined or unreliable sources. All or substantial proportion of cases diagnosed with poorly defined criteria from non‐clinical sources; positive predictive value judged to be poor; OR unable to determine
**Accuracy of comorbidity determination**
*Reviewer's judgment of the accuracy of diagnoses of comorbidity*:
1 = High – DSM‐based from specialist diagnosis or valid structured interview. Determined mainly from either structured interview designed to make DSM diagnosis, or diagnosis by a psychiatrist, when such data are judged to have good‐to‐excellent positive predictive value for the specific comorbidity of interest
2 = Good – not DSM‐based, but derived from good quality administrative data, non‐specialist diagnoses, or rating scales deemed to have good PPV (>70%). Determined largely or wholly from ICD, READ, or other diagnostic coding, when such data are judged to have good positive predictive value for the specific comorbidity of interest
3 = Fair – only self ‐report without a structured interview or fair administrative data. Determined largely or wholly from rating scales where a diagnosis is not made but a continuous measure used, patient report, or symptoms (reported or in the medical record) but not diagnosis when such data are judged to have only fair to good positive predictive value for the specific comorbidity of interest when such data are judged to have only fair positive predictive value for the specific comorbidity of interest
4 = Poor – ill‐defined or unreliable sources. Poor quality data or unable to determine

Abbreviations: DSM, Diagnostic and Statistical Manual of Mental Disorders; ICD, International Classification of Diseases; ILAE, International League Against Epilepsy. PPV, Positive Predictive Value; Numeric coding for ILAE overall ratings: 1 = High, 2 = Good, 3 = Fair, 4 = Poor. The overall ILAE rating is determined by the lowest‐scoring applicable domain.

### 2.4 Rating process and data extraction

Two independent raters (C.S.K. and A.R.) underwent standardized calibration using written scoring guidance and pilot ratings. Both reviewers had formal training in epilepsy epidemiology and systematic review methodology, with prior experience conducting epilepsy SRMAs. The order of tool application (NOS vs ILAE) and article assessment was randomized for each rater to minimize learning and order effects. All risk of bias ratings were conducted de novo for the present validation study and were not imported from prior SRMAs. Nevertheless, we acknowledge that rater expertise and prior familiarity with the literature may limit transferability to non‐expert users. Discrepancies were resolved by consensus or arbitration by a third reviewer (N.J. or C.R.N.). NOS scores and ILAE ratings were retrieved or calculated using extracted data from each SRMA.

### 2.5 Methodology and statistical analysis

Correlation between NOS and ILAE scores was assessed using Spearman's rank correlation.^8^ For this purpose, overall ILAE ratings were treated as ordinal scores and analyzed against NOS total scores using Spearman's rank correlation. Agreement between NOS and ILAE tools was assessed using Cohen's weighted kappa, using binarized overall ratings (Poor vs Higher‐than‐Poor) for both tools.^9^ Bland–Altman plots were used to identify systematic differences in numeric scores, reporting mean difference and upper and lower levels of agreement (LoAs).^10^ The LoAs were calculated as the mean difference ± 1.96 × the standard deviation (SD) of the differences, corresponding to the 95% range of expected variation between the two measurement methods. Bland–Altman plots displayed the difference between binarized ratings (both NOS and ILAE) against the mean of the original NOS and ILAE scores. This approach was used to assess whether systematic disagreement varied across the spectrum of underlying study quality rather than to evaluate categorical concordance. Test–retest reproducibility reflected repeated ratings by the same rater at a 6‐month interval, blinded to prior scores, using a two‐way mixed‐effects model with absolute agreement for single measurements.^11^ Original NOS total scores were analyzed on their native scales without rescaling, and non‐applicable ILAE domains did not contribute to overall ratings, as the lowest‐scoring applicable domain determined the final classification.

## 3 RESULTS

### 3.1 Study characteristics

A total of 54 studies were included. The NOS and ILAE assessments are available for all studies (see Table S1). Table 2 summarizes a comparison of the domains between the ILAE tool and NOS. Inter‐rater reliability of the ILAE Risk of Bias Tool has been previously reported for each of the three underlying SRMAs using Cohen's kappa (0.82–0.86), indicating excellent agreement; therefore, inter‐rater reliability was not re‐estimated in the present validation study.^3^, ^4^, ^5^

**TABLE 2:** Comparison of NOS and ILAE risk of bias tools.

Domain	NOS	ILAE
Source population	Selection domain	Explicitly rated as separate domain
Epilepsy case ascertainment	Exposure item	Completeness of case finding + diagnostic accuracy
Comorbidity ascertainment	Outcome item	Sensitivity + diagnostic rigor of comorbidities
Representativeness	Implicit in selection	Explicitly evaluated
Scoring structure	Numeric total (0–10 or 9)	Ordinal tier (High, Good, Fair, Poor)
Domain specificity	General epidemiology	Epilepsy‐specific validation criteria

### 3.2 Correlation between NOS and ILAE scores

**FIGURE 1:**
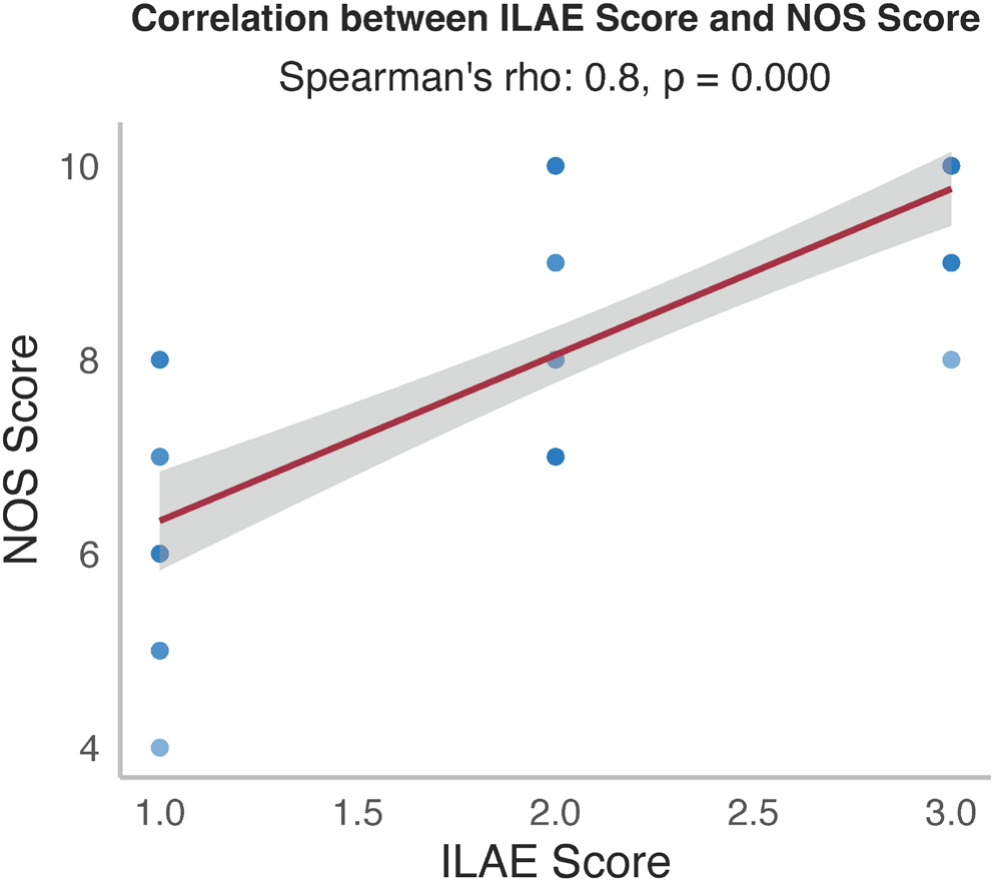
Scatterplot showing the correlation between International League Against Epilepsy (ILAE) and Newcastle–Ottawa Scale (NOS) scores.

There was a significant and strong positive correlation between total NOS scores and ILAE ratings (Spearman's rho = 0.80, 95% CI 0.68–89, *p* < 0.001), suggesting a shared directional pattern in their assessment of methodological quality (Figure 1).

### 3.3 Agreement across categorical tiers

To compare categorical agreement between NOS and ILAE tools, scores were collapsed into binary levels: Poor vs Higher‐than‐Poor for both tools. Cohen's weighted kappa was 0.68 (95% CI 0 0.37–0.92, *p* < 0.001), indicating substantial agreement in distinguishing low‐quality studies between the two tools.

### 3.4 Bland–Altman analysis

**FIGURE 2:**
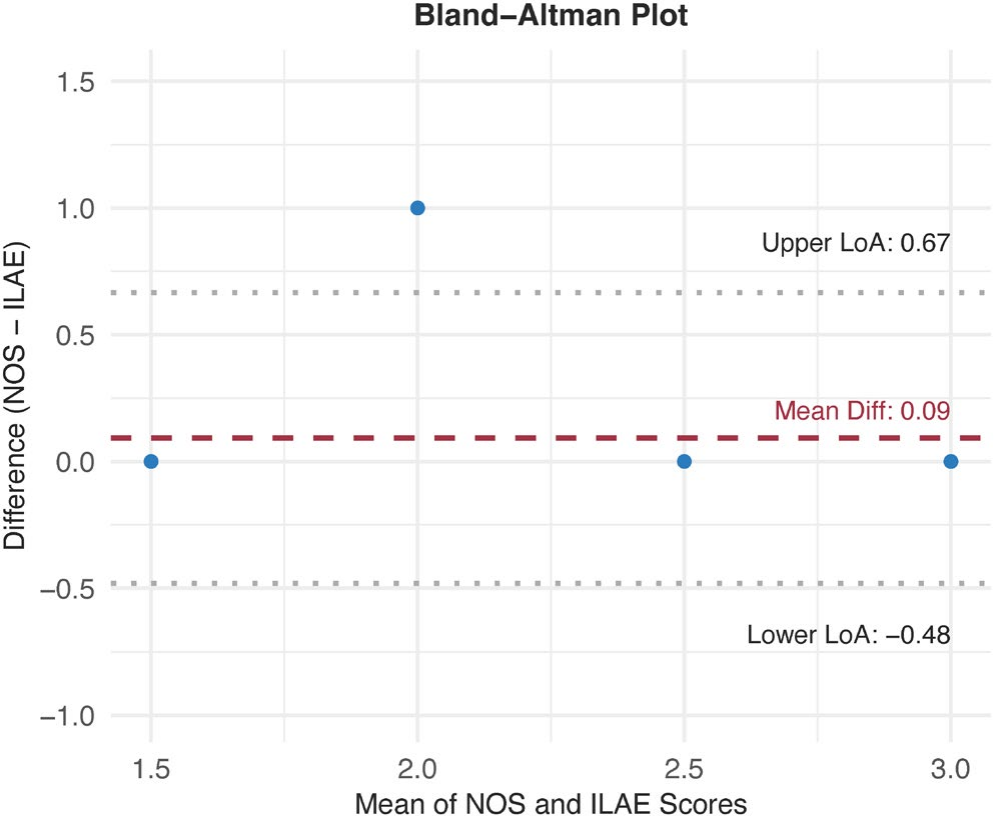
Bland–Altman plot comparing Newcastle‐Ottawa Scale (NOS) and International League Against Epilepsy (ILAE) Risk of Bias scores. LoAs, limits of agreement; Diff, difference; Bland–Altman plot showing the difference between binarized NOS and ILAE ratings (*y*‐axis) plotted against the mean of the original NOS and ILAE scores (*x*‐axis). Dashed line indicates the mean difference; dotted lines represent the 95% limits of agreement.

Bland–Altman analysis plotted the difference between binarized ratings (for both NOS and ILAE) against the mean of the original NOS and ILAE scores. The Bland–Altman plot (Figure 2) demonstrated a mean score difference of 0.09 (lower LoA: −0.48 – Upper LoA: 0.67) between NOS and ILAE numeric equivalents, based on binary levels: Poor vs Higher‐than‐Poor for both tools. These limits of agreement indicate minimal systematic bias across the range of underlying study quality, with NOS tending to assign slightly higher ratings on average compared with the more conservative ILAE criteria. In this context, the Bland–Altman plot evaluates whether disagreement varies across the spectrum of study quality rather than categorical concordance between tools.

### 3.5 The test–retest agreement of the ILAE tool

The findings demonstrated excellent test–retest reproducibility in the ILAE scoring assessment across different time points. The test–retest agreement between ILAE ratings at baseline (test) vs the 6‐month follow‐up (retest) was shown to be excellent with an intraclass correlation coefficient (ICC) of 0.90 (95% CI: 0.83–0.94).

## 4 DISCUSSION

This study assessed the reliability, validity, and reproducibility of the newly developed ILAE Risk of Bias Tool in comparison with the NOS, using a sample of observational studies in epilepsy research. The results indicate that the ILAE tool demonstrates excellent test–retest reproducibility, strong correlation with NOS scores, and substantial agreement in identifying low‐quality studies, while offering additional relevance through epilepsy‐specific domains.

Although the tools align well on average, they are not interchangeable. The ILAE tool incorporates epilepsy‐specific rigor, particularly in diagnostic accuracy and case‐finding, which are often only indirectly captured by NOS. The ILAE tool's explicit evaluation of epilepsy‐specific domains likely contributes to its tendency to assign more conservative ratings compared with general‐purpose tools such as the NOS. Of note, the use of a binary classification (Poor vs Higher‐than‐Poor) for Cohen's kappa allowed us to evaluate whether the tools reliably identify the most methodologically limited studies. The substantial kappa value suggests meaningful alignment in this regard, even as other results (e.g., Bland–Altman) showed that NOS may be more lenient overall. Together, these findings support the ILAE tool's reliability and its potential as an epilepsy‐specific alternative to the NOS for quality assessment in systematic reviews.

The ILAE tool demonstrated excellent 6‐month test–retest reproducibility, supported by a high ICC. The strong positive correlation between the two instruments suggests that they share a common framework for evaluating methodological quality. However, analysis using Bland–Altman plots revealed that NOS scores were, on average, slightly higher than those produced by the ILAE tool, indicating that the NOS is more lenient in certain areas of quality assessment.

Of note, the ILAE Risk of Bias Tool demonstrated strong reliability and agreement with the NOS, with the added benefit of epilepsy‐specific domains. These include explicit assessments of diagnostic accuracy, case‐finding sensitivity, and population representativeness, factors that are particularly relevant in epilepsy research but often not directly captured by general tools like the NOS. This structured, disease‐focused framework supports the use of the ILAE tool as an instrument for quality appraisal in future SRMAs focused on epilepsy.

Agreement between the two tools was also substantial when categorizing low‐quality studies. When ratings were collapsed into binary tiers (Poor vs Higher‐than‐Poor), the tools showed strong concordance, supporting the validity of the ILAE tool in identifying methodologically limited studies. This capability is particularly important in systematic reviews and meta‐analyses, where inclusion of lower‐quality studies may bias pooled estimates and compromise interpretability.

Several limitations should be acknowledged. The validation sample was restricted to three SRMAs of psychiatric comorbidities in epilepsy, predominantly involving observational designs, which may limit generalizability to other epilepsy research contexts, such as intervention studies, surgical series, or smaller clinical cohorts. Furthermore, although the NOS served as a benchmark for comparison, it is not without its limitations and should not be regarded as a gold standard. Although the use of two independent reviewers reflects standard practice in methodological validation studies, it may nonetheless limit the generalizability of reliability estimates beyond typical SRMA settings. Eligible studies required ≥30 participants per group to align with the inclusion criteria of the parent SRMAs; however, this threshold may have excluded smaller, potentially lower‐quality studies and influenced the distribution of risk‐of‐bias ratings. Binary categorization (Poor vs Higher‐than‐Poor) was used to focus on agreement in identifying studies at highest risk of bias, which most directly inform inclusion, exclusion, and sensitivity analyses in SRMAs. Distinguishing among higher‐quality tiers, although important, was beyond the primary scope of this analysis and warrants further investigation. The present evaluation was conducted using SRMAs addressing psychiatric comorbidities in epilepsy. Although the ILAE Risk of Bias Tool was developed for broader application across epilepsy comorbidity studies, its performance was examined here within this specific context. Finally, although this study supports the reliability and construct validity of the ILAE tool, future research should examine its performance across a broader range of epilepsy research questions and explore its impact on review outcomes and conclusions.

In summary, the ILAE Risk of Bias Tool offers a reliable, conceptually relevant, and field‐specific alternative to the NOS for assessing observational studies in epilepsy research. Widespread adoption could improve the methodological rigor and transparency of evidence synthesis in the field, ultimately strengthening the foundation on which clinical and policy decisions are made.

## AUTHOR CONTRIBUTIONS

**Churl‐Su Kwon:** conceptualization, formal analysis, investigation, data curation, methodology, visualization, writing of the original draft, project administration, supervision, validated, reviewed and edited the manuscript. **Ali Rafati:** formal analysis, investigation, data curation, methodology, visualization, writing of the original draft, validated, reviewed and edited the manuscript. **Nathalie Jette:** methodology, supervision, validated, reviewed and edited the manuscript. **Charles R. Newton:** methodology, supervision, validated, reviewed and edited the manuscript. All authors read and approved the final manuscript.

## CONFLICT OF INTEREST STATEMENT

The authors declare no conflicts of interest.

## ETHICS STATEMENT

We confirm that we have read the Journal's position on issues involved in ethical publication and affirm that this report is consistent with those guidelines.

## SOCIAL MEDIA SENTENCE

The ILAE Risk of Bias Tool shows strong reliability and agreement with the Newcastle–Ottawa Scale (NOS) in epilepsy systematic reviews and meta‐analyses (SRMAs).

## Supporting information


Table S1

## Data Availability

The data are available in the supplemental material.
