# Supplementary material for: Validation of the International League Against Epilepsy (ILAE) Risk of Bias Tool against the Newcastle–Ottawa Scale in epilepsy research
Source: Epilepsia. 2026 Mar 8;67(6):2890–6. doi: 10.1002/epi.70183 (PMC13285234; doi:10.1002/epi.70183)
Supplement: Supplementary file 1 — Table S1 [file EPI-67-2890-s001.docx]

**Supplemental Table- Methodological Quality Ratings of Included Studies by NOS and ILAE Tools**.

| **No.** | **Study** | **ILAE rating (Baseline)** | **ILAE rating (6-month FU)** | **NOS score** |
| --- | --- | --- | --- | --- |
|  | Uepping et al., 2021 | 2 | 2 | 10 |
|  | Kwon et al., 2020 | 2 | 2 | 10 |
|  | Wirrel et al., 2020 | 3 | 2 | 10 |
|  | Andersson et al., 2020 | 2 | 2 | 10 |
|  | Gorton et al., 2018 | 2 | 2 | 10 |
|  | Brikell et al., 2018 | 2 | 2 | 10 |
|  | Harnod et al., 2018, (Children) | 2 | 2 | 10 |
|  | Harnod et al., 2018, (Adults) | 2 | 2 | 10 |
|  | Weatherburn et al., 2017 | 2 | 2 | 10 |
|  | Gillberg et al., 2017 | 2 | 2 | 10 |
|  | Aaberg et al., 2016 | 2 | 2 | 10 |
|  | Jokiranta et al., 2014 | 3 | 2 | 10 |
|  | Fazel et al., 2013 | 2 | 2 | 10 |
|  | Rai et al., 2012 | 3 | 3 | 9 |
|  | Kessler et al., 2012 | 2 | 3 | 9 |
|  | Kwon et al., 2011 | 2 | 2 | 10 |
|  | Copeland et al., 2011 | 3 | 2 | 10 |
|  | Ettinger et al., 2010 | 2 | 2 | 10 |
|  | Stefanello et al., 2010 | 3 | 2 | 9 |
|  | Tellez-Zenteno et al., 2007 | 3 | 3 | 9 |
|  | Kobau et al., 2006 | 3 | 3 | 8 |
|  | Nuyen et al., 2006 | 3 | 2 | 10 |
|  | Gaitatizis et al., 2004 | 2 | 2 | 10 |
|  | Ettinger et al., 2005 | 3 | 3 | 9 |
|  | Ettinger et al., 2004 | 3 | 3 | 9 |
|  | Davies et al., 2003 | 2 | 2 | 8 |
|  | Stefansson et al., 1998 | 2 | 2 | 10 |
|  | Thomas et al., 2017 | 3 | 2 | 8 |
|  | Miniksar et al., 2022 | 3 | 3 | 7 |
|  | Almane et al., 2018 | 3 | 3 | 7 |
|  | Ekinci al., 2017 | 3 | 3 | 7 |
|  | Caplan et al., 2018 | 3 | 3 | 7 |
|  | Amruth et al., 2014 | 3 | 3 | 7 |
|  | Gulpek et al., 2011 | 3 | 3 | 7 |
|  | Ertekin et al., 2009 | 3 | 3 | 7 |
|  | Gupta et al., 2020 | 3 | 3 | 7 |
|  | Li et al., 2018 | 3 | 3 | 7 |
|  | Amiet et al., 2013 | 4 | 4 | 5 |
|  | Chung et al., 2013 | 4 | 4 | 5 |
|  | Cohen et al., 2012 | 4 | 4 | 6 |
|  | Filho et al., 2007 | 4 | 4 | 8 |
|  | Filho et al., 2010 | 4 | 4 | 8 |
|  | Hilger et al., 2013 | 4 | 4 | 4 |
|  | Tadokoro et al., 2007 | 4 | 4 | 6 |
|  | Salayev et al., 2017 | 4 | 4 | 7 |
|  | Yates et al., 2020 | 4 | 4 | 7 |
|  | Sorge et al., 2016 | 4 | 4 | 6 |
|  | Patten et al., 2018 | 4 | 4 | 8 |
|  | Carrozzino et al., 2016 | 4 | 4 | 6 |
|  | Erlangsen et al., 2020 | 2 | 2 | 10 |
|  | Hesdorffer et al., 2016 | 2 | 2 | 10 |
|  | Nevalainen et al., 2013 | 2 | 2 | 10 |
|  | Bornovski et al., 2021 | 2 | 2 | 10 |
|  | Arana et al., 2010 | 2 | 2 | 10 |

ILAE numeric coding: 1 = High, 2 = Good, 3 = Fair, 4 = Poor. Overall ratings are derived using the ILAE ‘worst-domain’ rule, whereby the lowest-scoring applicable domain determines the final rating. Non-applicable domains are excluded from this determination.

**References (studies included in the supplemental table)**

1. Uepping P, Hamer H, Scholten J, Kostev K. Physical and mental health comorbidities of patients with epilepsy in Germany - A retrospective cohort study. Epilepsy Behav. Apr 2021;117:107857. doi:10.1016/j.yebeh.2021.107857
2. Kwon CS, Wong B, Agarwal P, et al. Nonelective hospital admissions, discharge disposition, and health services utilization in epilepsy patients: A population-based study. Epilepsia. Sep 2020;61(9):1969-1978. doi:10.1111/epi.16642
3. Wirrell EC, Bieber ED, Vanderwiel A, Kreps S, Weaver AL. Self-injurious and suicidal behavior in young adults, teens, and children with epilepsy: A population-based study. Epilepsia. Sep 2020;61(9):1919-1930. doi:10.1111/epi.16618
4. Andersson K, Ozanne A, Edelvik Tranberg A, et al. Socioeconomic outcome and access to care in adults with epilepsy in Sweden: A nationwide cohort study. Seizure. Jan 2020;74:71-76. doi:10.1016/j.seizure.2019.12.001
5. Gorton HC, Webb RT, Carr MJ, DelPozo-Banos M, John A, Ashcroft DM. Risk of Unnatural Mortality in People With Epilepsy. JAMA Neurol. Aug 1 2018;75(8):929-938. doi:10.1001/jamaneurol.2018.0333
6. Brikell I, Ghirardi L, D'Onofrio BM, et al. Familial Liability to Epilepsy and Attention-Deficit/Hyperactivity Disorder: A Nationwide Cohort Study. Biol Psychiatry. Jan 15 2018;83(2):173-180. doi:10.1016/j.biopsych.2017.08.006
7. Harnod T, Lin CL, Kao CH. Prevalence of suicide attempts and their risk factors in school-aged patients with epilepsy: a population-based study. Eur Child Adolesc Psychiatry. Aug 2018;27(8):1047-1053. doi:10.1007/s00787-018-1118-z
8. Harnod T, Lin CL, Kao CH. Evaluating clinical risk factors for suicide attempts in patients with epilepsy. J Affect Disord. Mar 15 2018;229:79-84. doi:10.1016/j.jad.2017.12.048
9. Weatherburn CJ, Heath CA, Mercer SW, Guthrie B. Physical and mental health comorbidities of epilepsy: Population-based cross-sectional analysis of 1.5 million people in Scotland. Seizure. Feb 2017;45:125-131. doi:10.1016/j.seizure.2016.11.013
10. Gillberg C, Lundström S, Fernell E, Nilsson G, Neville B. Febrile Seizures and Epilepsy: Association With Autism and Other Neurodevelopmental Disorders in the Child and Adolescent Twin Study in Sweden. Pediatr Neurol. Sep 2017;74:80-86.e2. doi:10.1016/j.pediatrneurol.2017.05.027
11. Aaberg KM, Bakken IJ, Lossius MI, et al. Comorbidity and Childhood Epilepsy: A Nationwide Registry Study. Pediatrics. Sep 2016;138(3)doi:10.1542/peds.2016-0921
12. Jokiranta E, Sourander A, Suominen A, Timonen-Soivio L, Brown AS, Sillanpää M. Epilepsy among children and adolescents with autism spectrum disorders: a population-based study. J Autism Dev Disord. Oct 2014;44(10):2547-57. doi:10.1007/s10803-014-2126-6
13. Fazel S, Wolf A, Långström N, Newton CR, Lichtenstein P. Premature mortality in epilepsy and the role of psychiatric comorbidity: a total population study. Lancet. Nov 16 2013;382(9905):1646-54. doi:10.1016/s0140-6736(13)60899-5
14. Rai D, Kerr MP, McManus S, Jordanova V, Lewis G, Brugha TS. Epilepsy and psychiatric comorbidity: a nationally representative population-based study. Epilepsia. Jun 2012;53(6):1095-103. doi:10.1111/j.1528-1167.2012.03500.x
15. Kessler RC, Lane MC, Shahly V, Stang PE. Accounting for comorbidity in assessing the burden of epilepsy among US adults: results from the National Comorbidity Survey Replication (NCS-R). Mol Psychiatry. Jul 2012;17(7):748-58. doi:10.1038/mp.2011.56
16. Kwon C, Liu M, Quan H, Thoo V, Wiebe S, Jetté N. Motor vehicle accidents, suicides, and assaults in epilepsy: a population-based study. Neurology. Mar 1 2011;76(9):801-6. doi:10.1212/WNL.0b013e31820e7b3b
17. Copeland LA, Ettinger AB, Zeber JE, Gonzalez JM, Pugh MJ. Psychiatric and medical admissions observed among elderly patients with new-onset epilepsy. BMC Health Serv Res. Apr 19 2011;11:84. doi:10.1186/1472-6963-11-84
18. Ettinger AB, Copeland LA, Zeber JE, Van Cott AC, Pugh MJ. Are psychiatric disorders independent risk factors for new-onset epilepsy in older individuals? Epilepsy Behav. Jan 2010;17(1):70-4. doi:10.1016/j.yebeh.2009.10.010
19. Stefanello S, Marín-Léon L, Fernandes PT, Li LM, Botega NJ. Psychiatric comorbidity and suicidal behavior in epilepsy: a community-based case-control study. Epilepsia. Jul 2010;51(7):1120-5. doi:10.1111/j.1528-1167.2009.02386.x
20. Tellez-Zenteno JF, Patten SB, Jetté N, Williams J, Wiebe S. Psychiatric comorbidity in epilepsy: a population-based analysis. Epilepsia. Dec 2007;48(12):2336-44. doi:10.1111/j.1528-1167.2007.01222.x
21. Kobau R, Gilliam F, Thurman DJ. Prevalence of self-reported epilepsy or seizure disorder and its associations with self-reported depression and anxiety: results from the 2004 HealthStyles Survey. Epilepsia. Nov 2006;47(11):1915-21. doi:10.1111/j.1528-1167.2006.00612.x
22. Nuyen J, Schellevis FG, Satariano WA, et al. Comorbidity was associated with neurologic and psychiatric diseases: a general practice-based controlled study. J Clin Epidemiol. Dec 2006;59(12):1274-84. doi:10.1016/j.jclinepi.2006.01.005
23. Gaitatzis A, Carroll K, Majeed A, J WS. The epidemiology of the comorbidity of epilepsy in the general population. Epilepsia. Dec 2004;45(12):1613-22. doi:10.1111/j.0013-9580.2004.17504.x
24. Ettinger AB, Reed ML, Goldberg JF, Hirschfeld RM. Prevalence of bipolar symptoms in epilepsy vs other chronic health disorders. Neurology. Aug 23 2005;65(4):535-40. doi:10.1212/01.wnl.0000172917.70752.05
25. Ettinger A, Reed M, Cramer J. Depression and comorbidity in community-based patients with epilepsy or asthma. Neurology. Sep 28 2004;63(6):1008-14. doi:10.1212/01.wnl.0000138430.11829.61
26. Davies S, Heyman I, Goodman R. A population survey of mental health problems in children with epilepsy. Dev Med Child Neurol. May 2003;45(5):292-5. doi:10.1017/s0012162203000550
27. Stefansson SB, Olafsson E, Hauser WA. Psychiatric morbidity in epilepsy: a case controlled study of adults receiving disability benefits. J Neurol Neurosurg Psychiatry. Feb 1998;64(2):238-41. doi:10.1136/jnnp.64.2.238
28. Thomas S, Hovinga ME, Rai D, Lee BK. Brief Report: Prevalence of Co-occurring Epilepsy and Autism Spectrum Disorder: The U.S. National Survey of Children's Health 2011-2012. J Autism Dev Disord 2017;47:224-229
29. Yıldız Miniksar D, Kılıç B, Kılıç M, Miniksar ÖH, Topçu Y, Aydın K. Evaluation of suicide probability in children and adolescents with epilepsy. Pediatr Int. 2022;64(1):e15130. doi:10.1111/ped.15130
30. Almane DN, Jones JE, McMillan T, et al. The Timing, Nature, and Range of Neurobehavioral Comorbidities in Juvenile Myoclonic Epilepsy. Pediatr Neurol. 2019;101:47-52. doi:10.1016/j.pediatrneurol.2019.03.011
31. Ekinci O, Okuyaz Ç, Gunes S, et al. Sleep problems in pediatric epilepsy and ADHD: The impact of comorbidity. Epilepsy Behav. 2017;71(Pt A):7-12. doi:10.1016/j.yebeh.2017.03.026
32. Caplan R, Siddarth P, Stahl L, et al. Childhood absence epilepsy: behavioral, cognitive, and linguistic comorbidities. Epilepsia. 2008;49(11):1838-1846. doi:10.1111/j.1528-1167.2008.01680.x
33. Amruth G, Praveen-Kumar S, Nataraju B, Kasturi P. Study of psychiatric comorbidities in epilepsy by using the Mini International Neuropsychiatric Interview. Epilepsy Behav. 2014;33:94-100. doi:10.1016/j.yebeh.2014.02.001
34. Gülpek D, Bolat E, Mete L, Arici S, Celebisoy M. Psychiatric comorbidity, quality of life and social support in epileptic patients. Nord J Psychiatry. 2011;65(6):373-380. doi:10.3109/08039488.2011.565798
35. Ertekin BA, Kulaksizoğlu IB, Ertekin E, et al. A comparative study of obsessive-compulsive disorder and other psychiatric comorbidities in patients with temporal lobe epilepsy and idiopathic generalized epilepsy. Epilepsy Behav. 2009;14(4):634-639. doi:10.1016/j.yebeh.2009.01.016
36. Gupta R, Garg D, Kumar N, et al. Psychiatric co-morbidities and factors associated with psychogenic non-epileptic seizures: a case-control study. Seizure. 2020;81:325-331. doi:10.1016/j.seizure.2020.05.007
37. Li T, Zhou H, Li Y, et al. Assessment of the neuropsychiatric comorbidities in Chinese children with epilepsy using the MINI-KID tool. Epilepsy Res. 2018;140:8-14. doi:10.1016/j.eplepsyres.2017.11.011
38. Amiet C, Gourfinkel-An I, Laurent C, et al. Epilepsy in simplex autism pedigrees is much lower than the rate in multiplex autism pedigrees. Biol Psychiatry. 2013;74(3):e3-e4. doi:10.1016/j.biopsych.2013.01.037
39. Chung MC, Allen RD, Dennis I. The impact of self-efficacy, alexithymia and multiple traumas on posttraumatic stress disorder and psychiatric co-morbidity following epileptic seizures: a moderated mediation analysis. Psychiatry Res. 2013;210(3):1033-1041. doi:10.1016/j.psychres.2013.07.041
40. Cohen R, Senecky Y, Shuper A, et al. Prevalence of epilepsy and attention-deficit hyperactivity (ADHD) disorder: a population-based study. J Child Neurol. 2013;28(1):120-123. doi:10.1177/0883073812440327
41. de Araújo Filho GM, Pascalicchio TF, Sousa Pda S, Lin K, Ferreira Guilhoto LM, Yacubian EM. Psychiatric disorders in juvenile myoclonic epilepsy: a controlled study of 100 patients. Epilepsy Behav. 2007;10(3):437-441. doi:10.1016/j.yebeh.2007.01.016
42. Filho GM, Jackowski AP, Lin K, et al. The integrity of corpus callosum and cluster B personality disorders: a quantitative MRI study in juvenile myoclonic epilepsy. Prog Neuropsychopharmacol Biol Psychiatry. 2010;34(3):516-521. doi:10.1016/j.pnpbp.2010.02.009
43. Hilger E, Zimprich F, Jung R, Pataraia E, Baumgartner C, Bonelli S. Postictal psychosis in temporal lobe epilepsy: a case-control study. Eur J Neurol. 2013;20(6):955-961. doi:10.1111/ene.12125
44. Tadokoro Y, Oshima T, Kanemoto K. Interictal psychoses in comparison with schizophrenia--a prospective study. Epilepsia. 2007;48(12):2345-2351. doi:10.1111/j.1528-1167.2007.01230.x
45. Salayev KA, Sanne B, Salayev R. Psychiatric and Behavioural Problems in Children and Adolescents with Epilepsy. East Asian Arch Psychiatry. 2017;27(3):106-114.
46. Yates K, Lång U, DeVylder J, et al. Prevalence and psychopathologic significance of hallucinations in individuals with a history of seizures. Epilepsia. 2020;61(7):1464-1471. doi:10.1111/epi.16570
47. Sorge ST, Hesdorffer DC, Phelan JC, et al. Depression and genetic causal attribution of epilepsy in multiplex epilepsy families. Epilepsia. 2016;57(10):1643-1650. doi:10.1111/epi.13500
48. Patten SB, Williams JVA, Lavorato DH, et al. Patterns of association of chronic medical conditions and major depression. Epidemiol Psychiatr Sci. 2018;27(1):42-50. doi:10.1017/S204579601600072X
49. Carrozzino D, Marchetti D, Laino D, et al. Anxiety in adolescent epilepsy. A clinimetric analysis. Nord J Psychiatry. 2016;70(6):424-429. doi:10.3109/08039488.2016.1143029
50. Erlangsen A, Stenager E, Conwell Y, et al. Association Between Neurological Disorders and Death by Suicide in Denmark. JAMA. 2020;323(5):444-454. doi:10.1001/jama.2019.21834
51. Hesdorffer DC, Ishihara L, Webb DJ, Mynepalli L, Galwey NW, Hauser WA. Occurrence and Recurrence of Attempted Suicide Among People With Epilepsy. JAMA Psychiatry. 2016;73(1):80-86. doi:10.1001/jamapsychiatry.2015.2516
52. Nevalainen O, Raitanen J, Ansakorpi H, Artama M, Isojärvi J, Auvinen A. Long-term mortality risk by cause of death in newly diagnosed patients with epilepsy in Finland: a nationwide register-based study. Eur J Epidemiol. 2013;28(12):981-990. doi:10.1007/s10654-013-9848-1
53. Bornovski Y, Jackson-Shaheed E, Argraves S, et al. Suicide and Seizures: A National Cohort Study in Veterans. Neurol Clin Pract. 2021;11(5):372-376. doi:10.1212/CPJ.0000000000001070
54. Arana A, Wentworth CE, Ayuso-Mateos JL, Arellano FM. Suicide-related events in patients treated with antiepileptic drugs. N Engl J Med. 2010;363(6):542-551. doi:10.1056/NEJMoa0909801
